# Supplementary material for: Phytochemical Analysis of Anastatica hierochuntica and Aerva javanica Grown in Qatar: Their Biological Activities and Identification of Some Active Ingredients
Source: Molecules. 2023 Apr 11;28(8):3364. doi: 10.3390/molecules28083364 (PMC10145884; doi:10.3390/molecules28083364)
Supplement: Supplementary file 1 [file molecules-28-03364-s001.zip › molecules-2307496-supplementary.pdf]

Supplementary Materials

Phytochemical Analysis of *Anastatica hierochuntica* and *Aerva javanica* Grown in Qatar: Their Biological Activities and Identification of Active Ingredients.

Vandana Thotathil, Naheed Sidiq, Ameena Fakroo and Lakshmaiah Sreerama*

Department of Chemistry and Earth Sciences, Chemistry Program, College of Arts and Sciences, Qatar University, P.O. Box 2713, Doha, Qatar
* Correspondence: author: lsreerama@qu.edu.qa

Anastatica Hierochuntica

Phytochemical analysis of the Isolated Compounds

The hexane fraction was subjected to HPTLC and HPLC analysis. Three pure compounds were isolated and subjected to IR and NMR analysis for further identification.

COMPOUND 1 (AH-HX-001-P1)

SPECIFICATON	SPECTRUM
Probable structure	β -Sitosterol 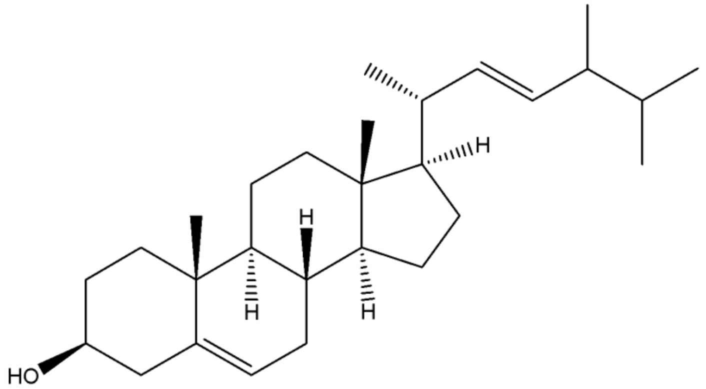
CAMAG HPTLC Scanner III<br>Mobile Phase: Hex: EtoAc (7:3)<br>Rf: 0.74<br>λ max: 203nm	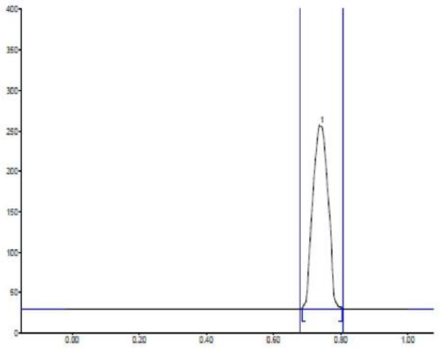 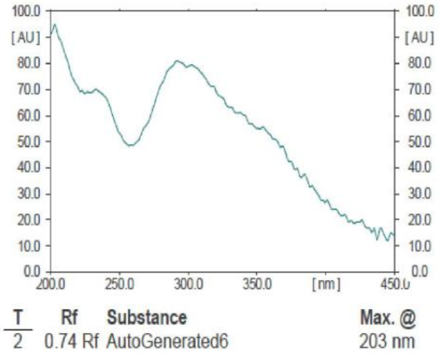
UV spectrum<br>PDA detector: 200-800nm<br>λ max: 194 nm	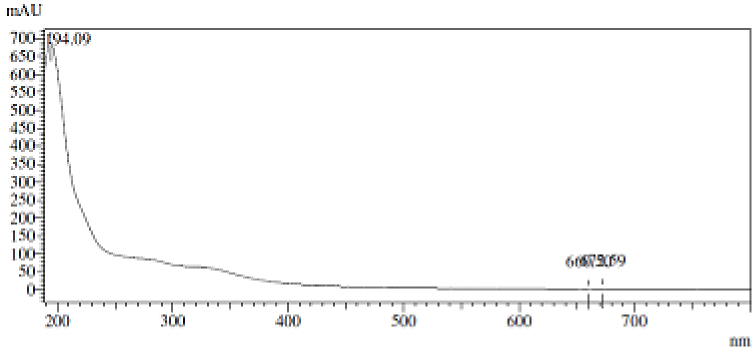

SHIMADZU SPD-20A HPLC<br>Column: Enable C_{18}, 4.6x250mm (5micron)<br>Mobile Phase: ACN:H_2O (15:85) with 0.1% H_3PO_4<br>λ max: 280nm<br>Inj. Vol: 20µl<br>Flow rate: 1.0ml/min<br>Rt: 5.339 min	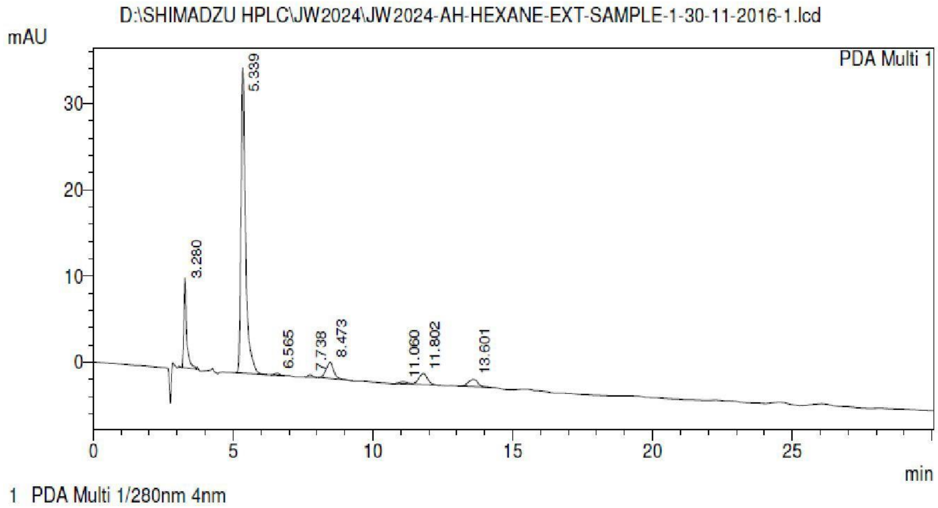
Bruker FT-IR<br>Wavelength scan: 500-4000cm^{-1}	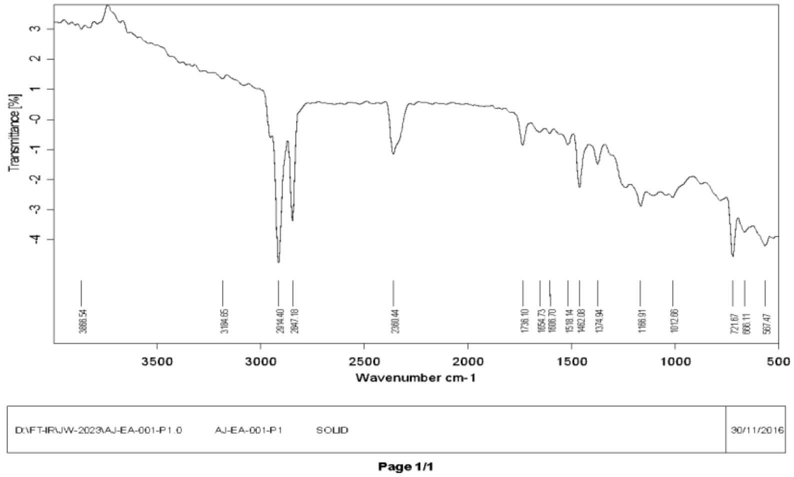
BRUKER NMR 400MHz<br>^{1}HNMR($CDCl_3$):	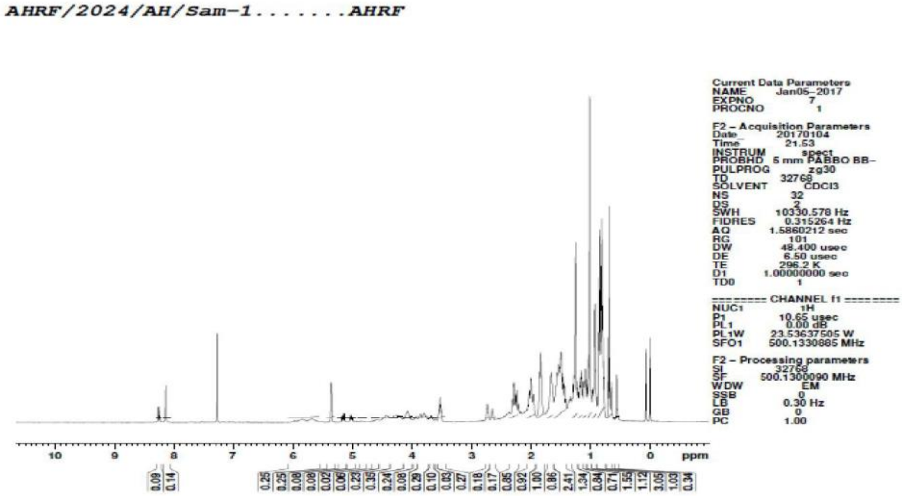

BRUKER NMR 400MHz ^{13}CNMR($CDCl_3$):

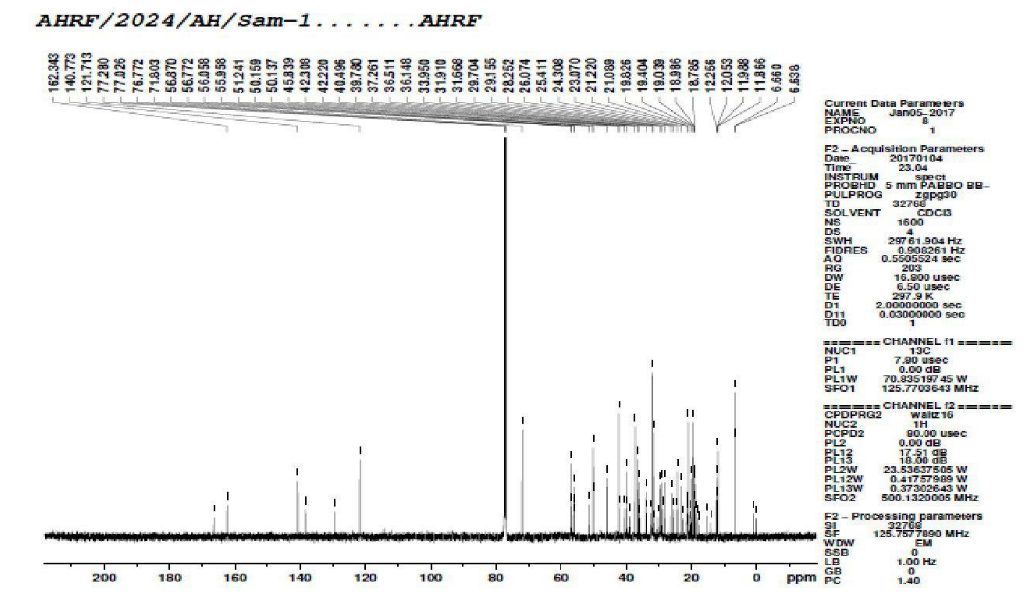

COMPOUND 2 (AH-HX-001-P4)

SPECIFICATON	SPECTRUM
Probable structure	Campesterol<br>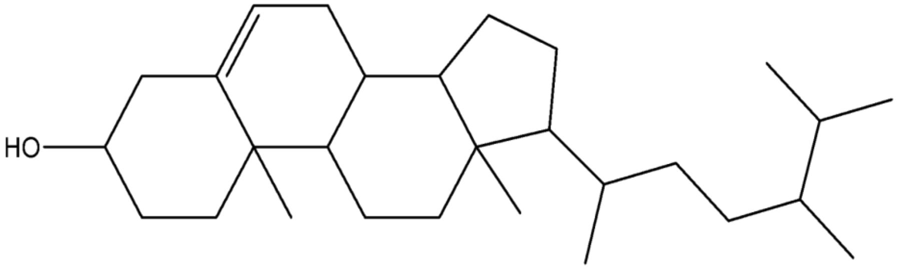
CAMAG HPTLC Scanner III<br>Mobile Phase: Hex: EtoAc (7:3)<br>Rf: 0.93<br>λ max: 202 nm	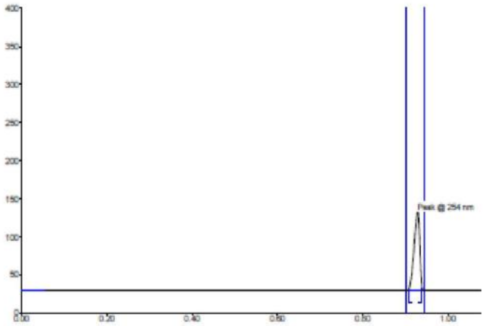<br>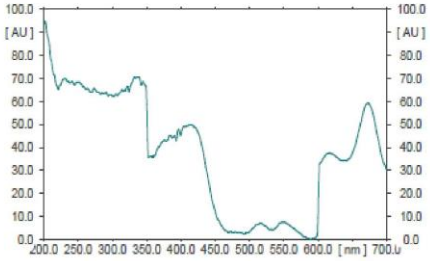<br><br>T Rf Substance Max. @<br>1 0.93 Rf Peak 202 nm
UV spectrum<br>PDA detector: 200-800nm<br>λ max: 205, 268nm	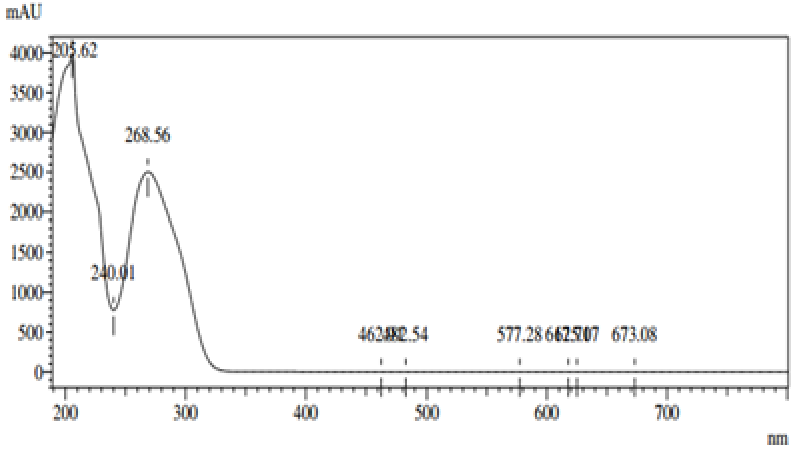

SHIMADZU SPD-20A HPLC<br>Column: Enable C_{18}, 4.6x250mm (5micron)<br>Mobile Phase: ACN:H_2O (15:85) with 0.1% H_3PO_4<br>λ max: 280nm<br>Inj. Vol: 20µl<br>Flow rate: 1.0ml/min<br>Rt: 4.914 min	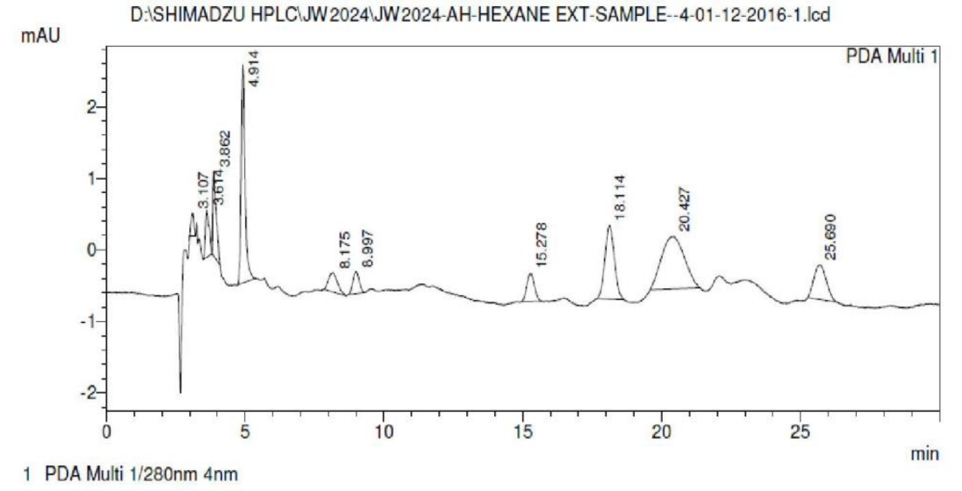
Bruker FT-IR<br>Wavelength scan: 500-4000cm^{-1}	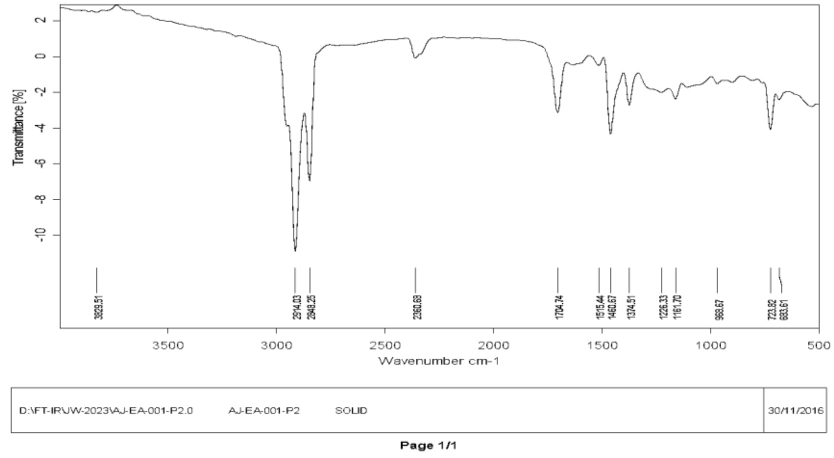
BRUKER NMR 400MHz<br>^{1}HNMR($CDCl_3$):	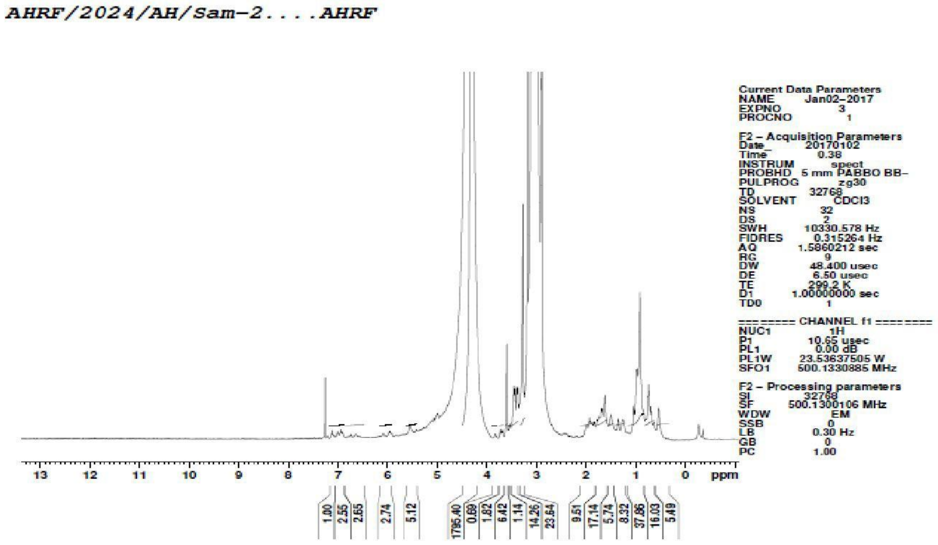

<table>
<tr>
<td>BRUKER NMR 400MHz
^{13}CNMR($CDCl_3$):</td>
<td>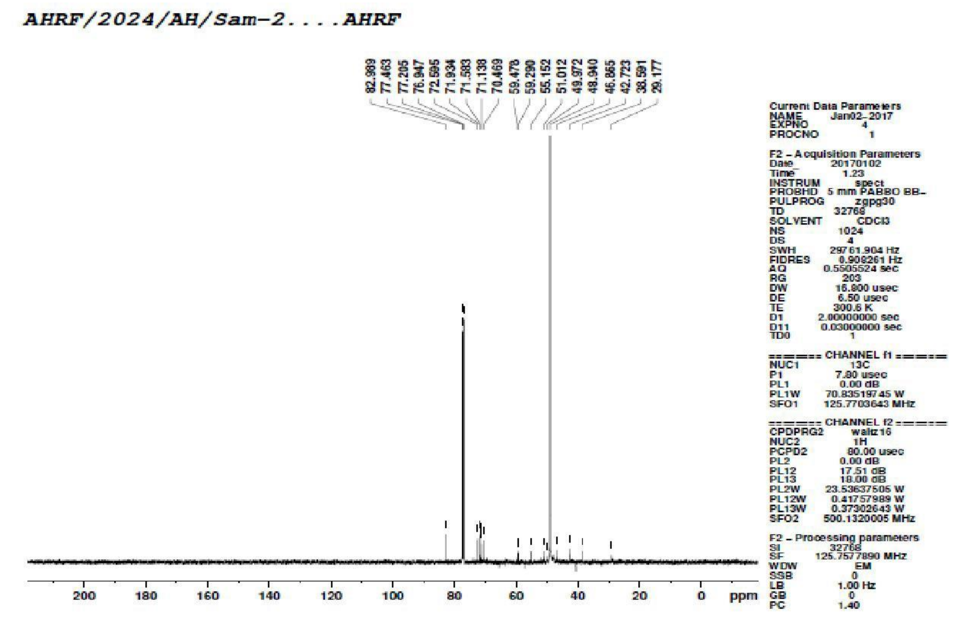
</td>
</tr>
</table>

COMPOUND 3 (AH-HX-001-P5)

<table>
<tr><th>SPECIFICATON</th><th>SPECTRUM</th></tr>
<tr><td>Probable structure</td><td>Methyl- 9-(4-(3,4-dihydroxy-1'-methyl-5'-oxocyclohexyl)-2-hydroxycyclohexyl)nonanoate
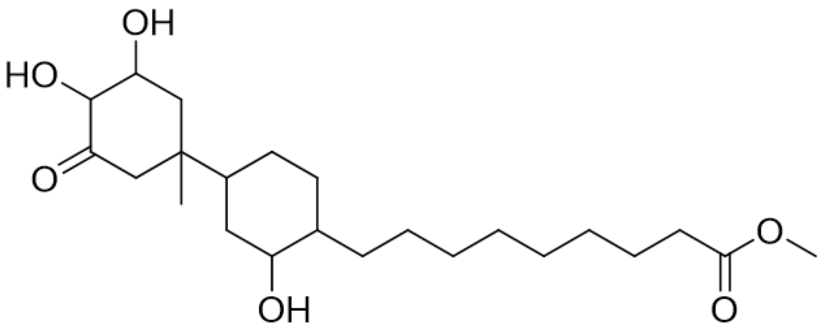</td></tr>
<tr><td>UV spectrum
PDA detector: 200-800nm
λ max: 205, 265nm</td><td>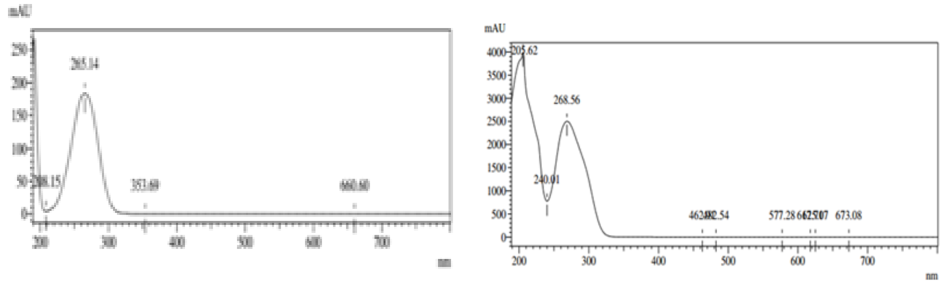
</td></tr>
<tr><td>CAMAG HPTLC Scanner III
Mobile Phase: Hex: EtoAc (7:3)
Rf: 0.09
λ max: 201 nm</td><td>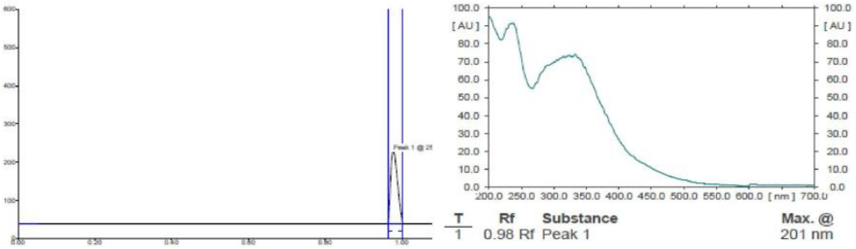
</td></tr>
</table>

SHIMADZU SPD-20A HPLC<br>Column: Enable C_{18}, 4.6x250mm (5micron)<br>Mobile Phase: ACN:H_2O (15:85) with 0.1% H_3PO_4<br>λ max: 280nm<br>Inj. Vol: 20µl<br>Flow rate: 1.0ml/min<br>Rt: 3.808min and 4.868min	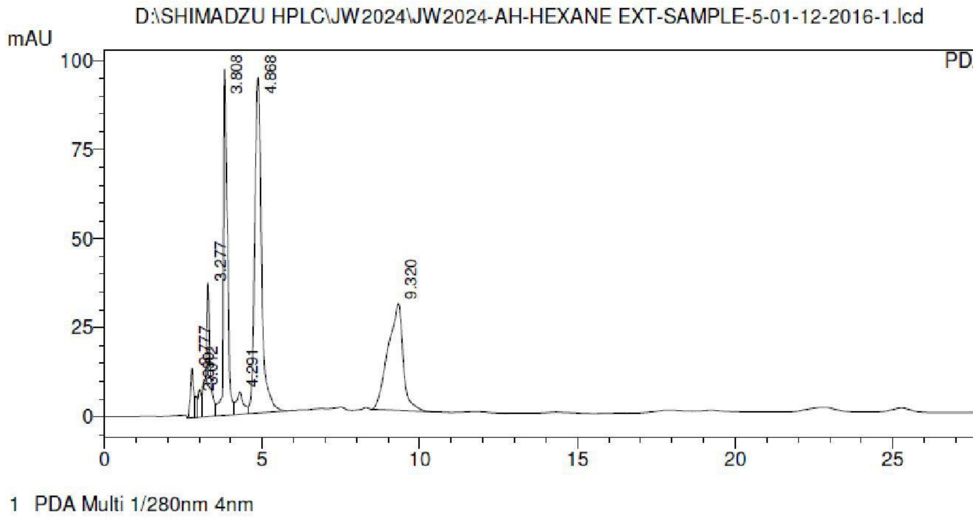

<table>
<tr><td>Bruker FT-IR
Wavelength scan:
500-4000cm^{-1}</td><td>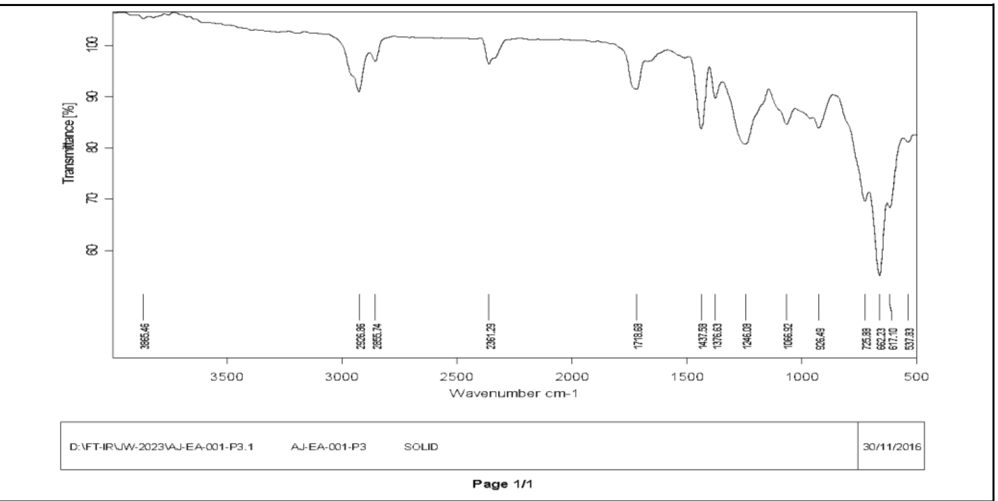
</td></tr>
<tr><td>BRUKER NMR 400MHz
^{1}HNMR($CDCl_3$):</td><td>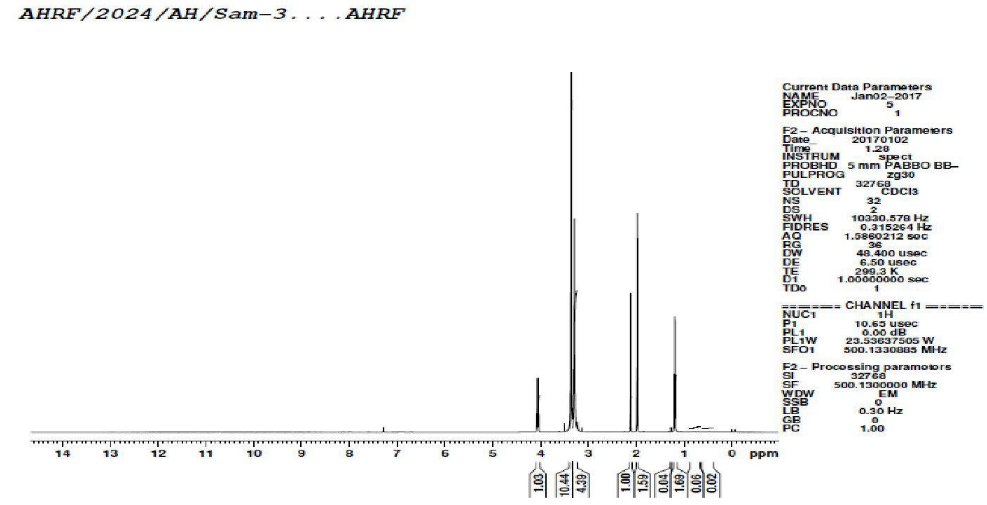
</td></tr>
<tr><td>BRUKER NMR 400MHz
^{13}CNMR($CDCl_3$):</td><td>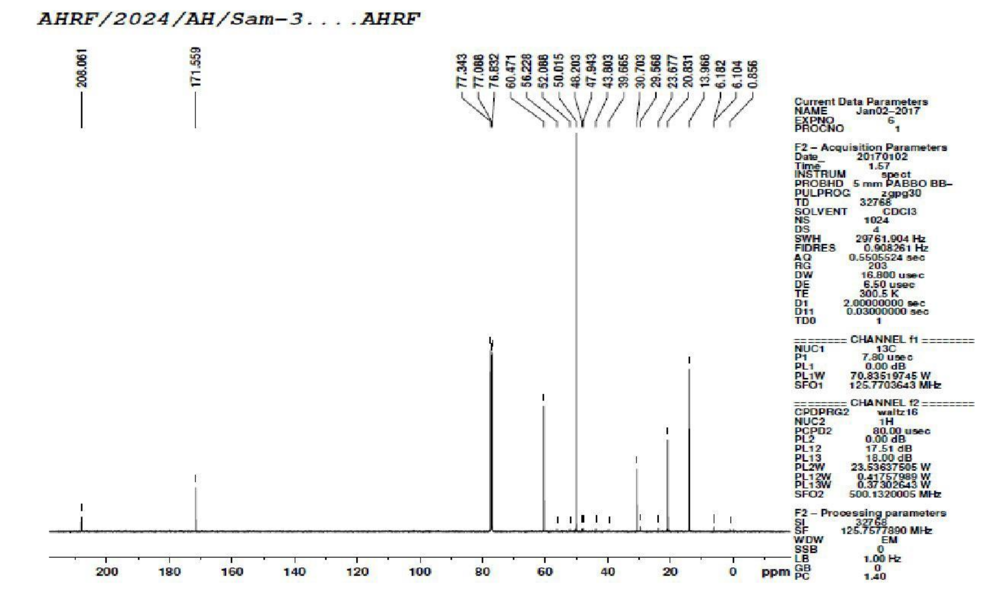
</td></tr>
</table>

Aerva Javanica

Phytochemical analysis of the Isolated Compounds

The ethyl acetate fraction was subjected to HPTLC and HPLC analysis. Three pure compounds were isolated and subjected to IR and NMR analysis for further identification.

COMPOUND 1 (AJ-EA-001-P1)

Probable structure	Lupenone 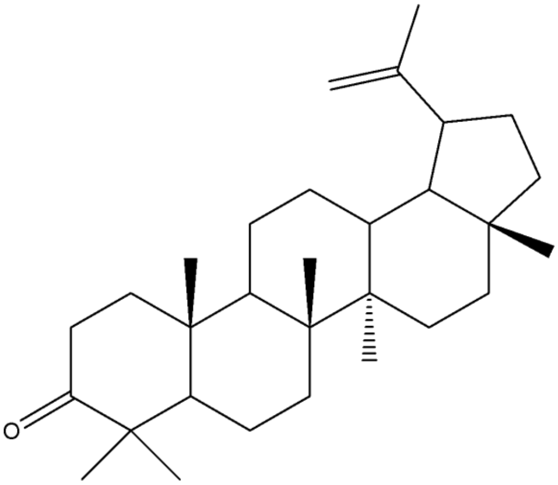
CAMAG HPTLC Scanner III<br>Mobile Phase: Hex: EtoAc (7:3)<br>Rf: 0.09<br>λ max: 254nm	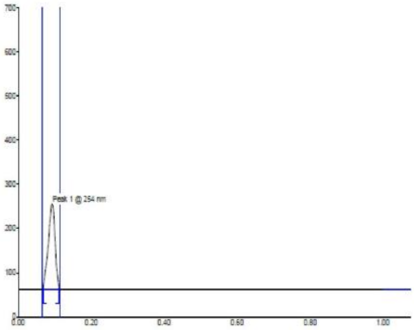 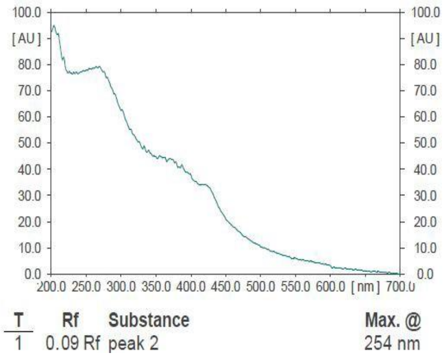

<table>
<tr>
<td>UV spectrum
PDA detector: 200-800nm
λ max: 205, 268nm</td>
<td>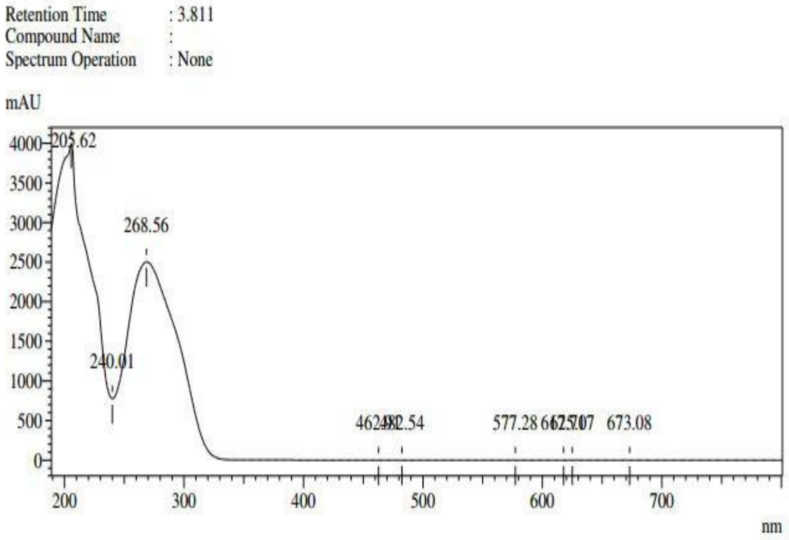
</td>
</tr>
<tr>
<td>SHIMADZU SPD-20A HPLC
Column: Enable C_{18}, 4.6x250mm (5micron)
Mobile Phase: ACN:H_2O (15:85) with 0.1% H_3PO_4
λ max: 280nm
Inj. Vol: 20µl
Flow rate: 1.0ml/min
Rt: 3.811min</td>
<td>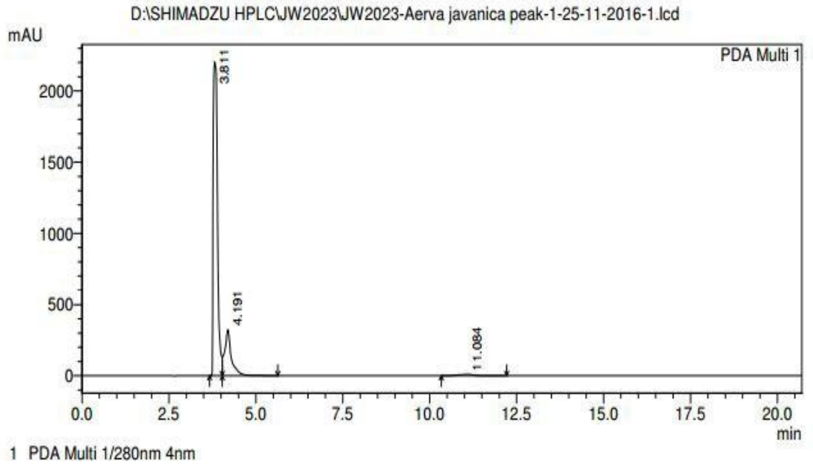
</td>
</tr>
<tr>
<td>Bruker FT-IR
Wavelength scan: 500-4000cm^{-1}</td>
<td>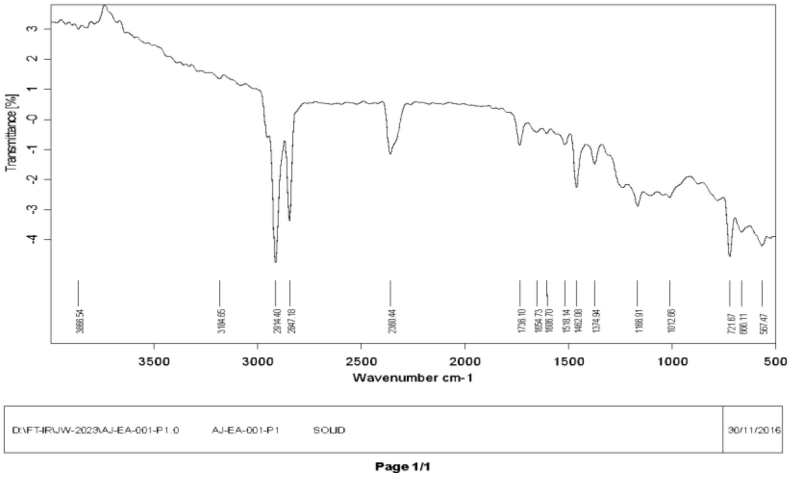
</td>
</tr>
</table>

BRUKER NMR 400MHz
^{1}HNMR($CDCl_3$):

AHRF/2023/AJ/Sam-1........Prince

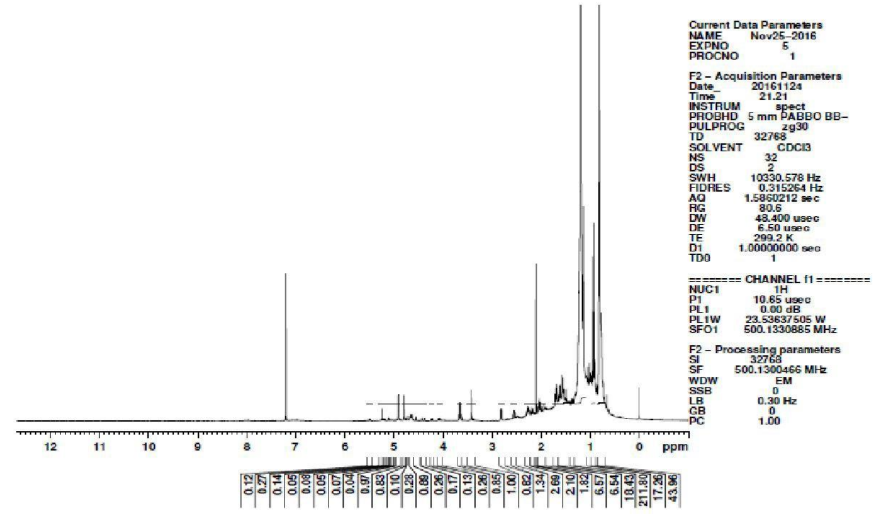

BRUKER NMR 400MHz
^{13}CNMR($CDCl_3$):

AHRF/2023/AJ/Sam-1........Prince

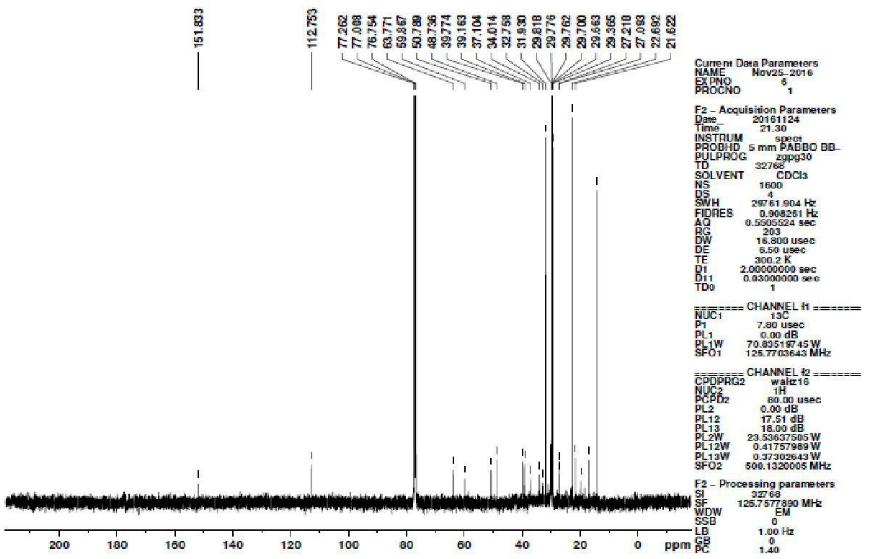

COMPOUND 2 (AJ-EA-001-P2)

SPECIFICATON	SPECTRUM
Probable structure	Betulinic acid<br>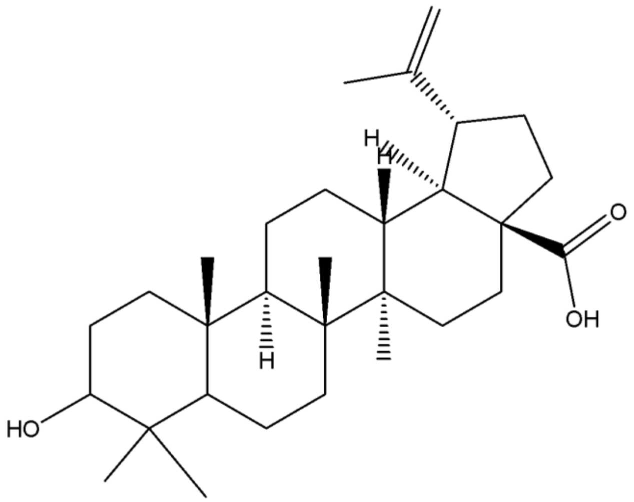
CAMAG HPTLC Scanner III<br>Mobile Phase: Hex: EtoAc (7:3)<br>Rf: 0.51<br>λ max: 204nm	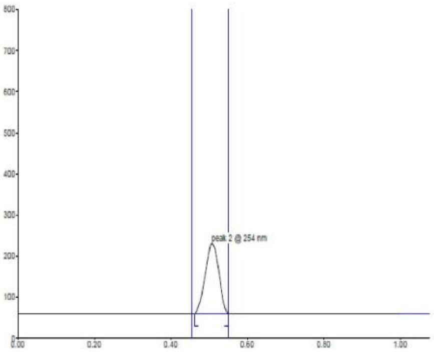<br><br>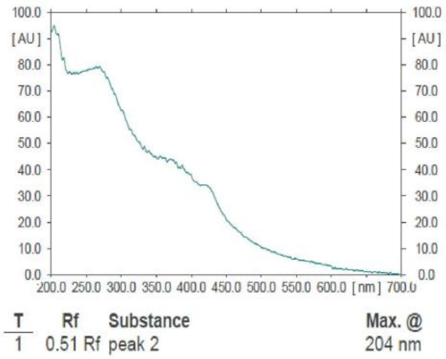
UV spectrum<br>PDA detector<br>λ max: Rt 3.814 – 215, 272nm<br>Rt 4.778 – 265nm	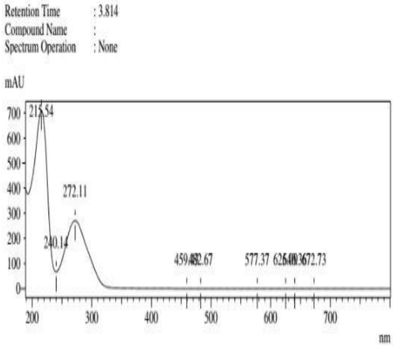<br><br>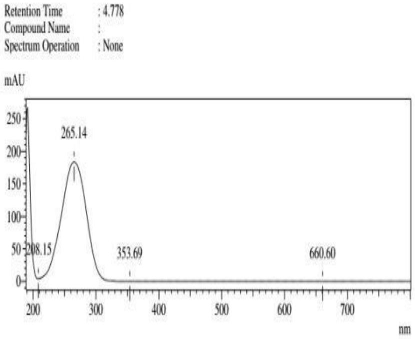

SHIMADZU SPD-20A HPLC<br>Column: Enable C_{18}, 4.6x250mm (5micron)<br>Mobile Phase: ACN:H_2O (15:85) with 0.1% H_3PO_4<br>λ max: 280nm<br>Inj. Vol: 20µl<br>Flow rate: 1.0ml/min<br>Rt: 3.811min and 4.778min	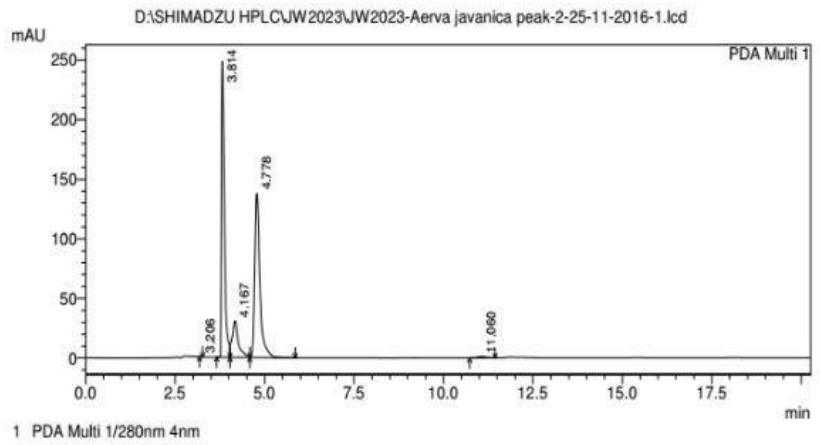
Bruker FT-IR<br>Wavelength scan: 500-4000cm^{-1}	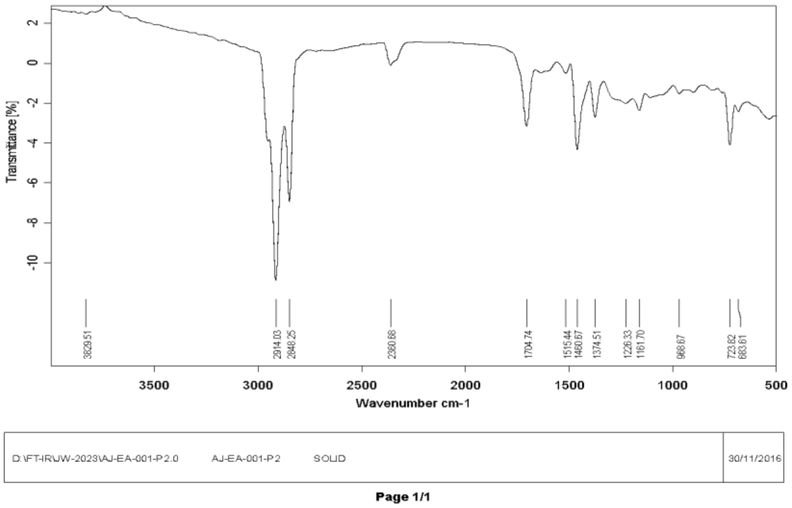
BRUKER NMR 400MHz<br>^{1}HNMR($CDCl_3$):	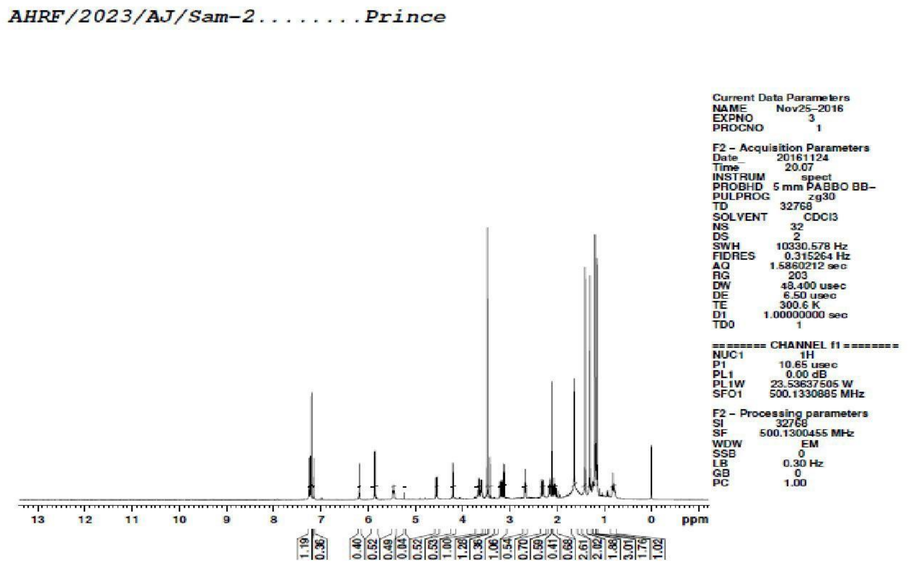

BRUKER NMR 400MHz<br>13CNMR($CDCl_3$):	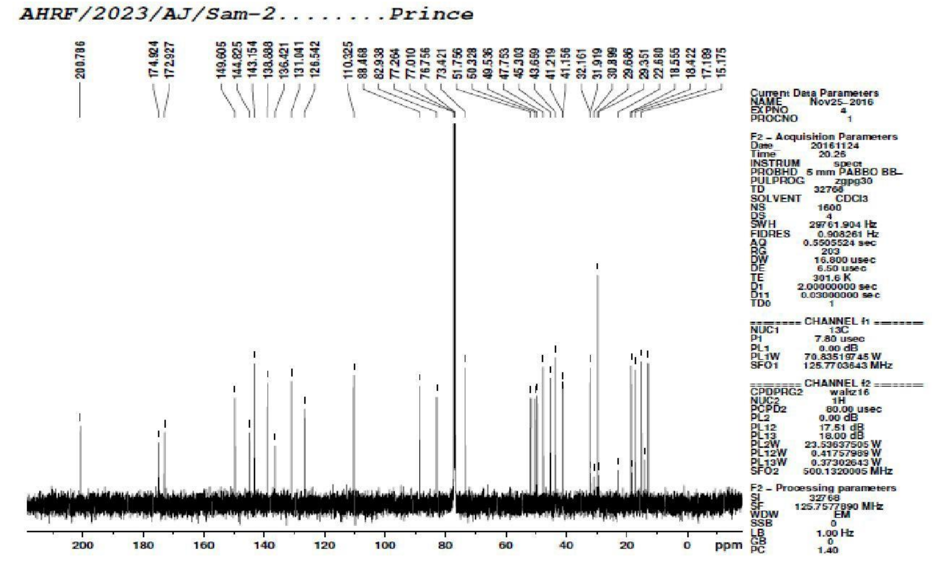

COMPOUND 3 (AJ-EA-001-P6)

SPECIFICATON	SPECTRUM
Probable structure	Lupeol acetate 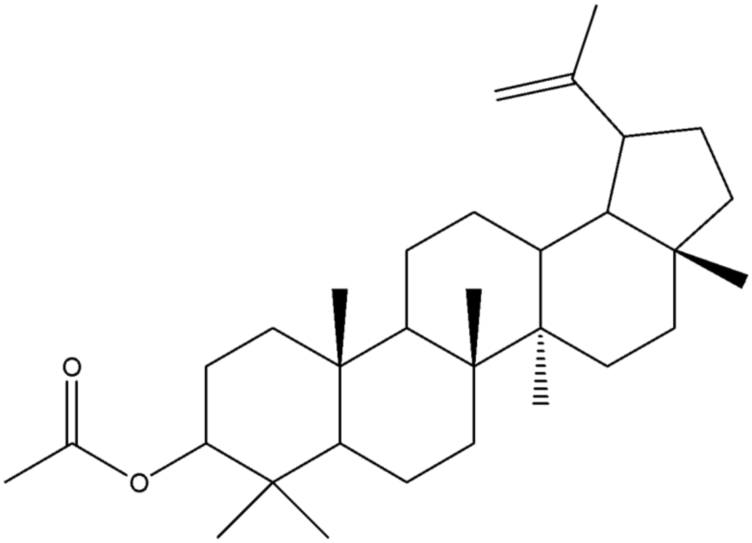
CAMAG HPTLC Scanner III<br>Mobile Phase: Hex: EtoAc (7:3)<br>Rf: 0.68<br>λ max: 254nm	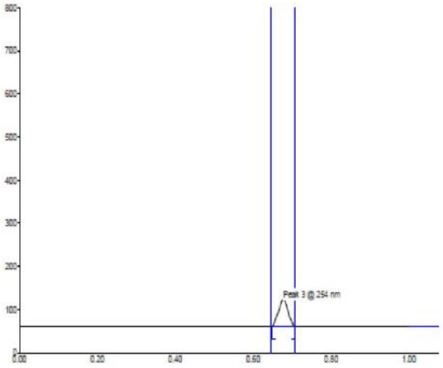 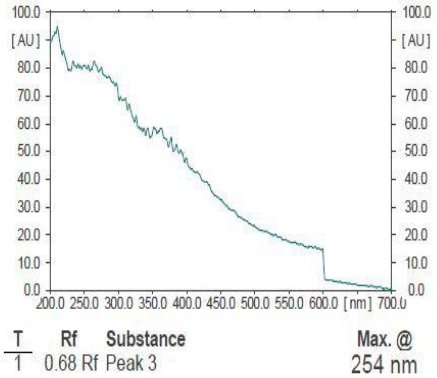
UV spectrum<br>PDA detector<br>λ max: 265nm	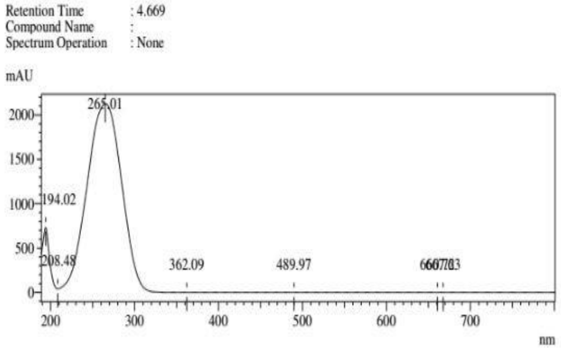

<table>
<tr>
<td>SHIMADZU SPD-20A HPLC
Column: Enable C_{18}, 4.6x250mm (5micron)
Mobile Phase: ACN:H_2O (15:85) with 0.1% H_3PO_4
λ max: 280nm
Inj. Vol: 20µl
Flow rate: 1.0ml/min
Rt: 4.669min</td>
<td>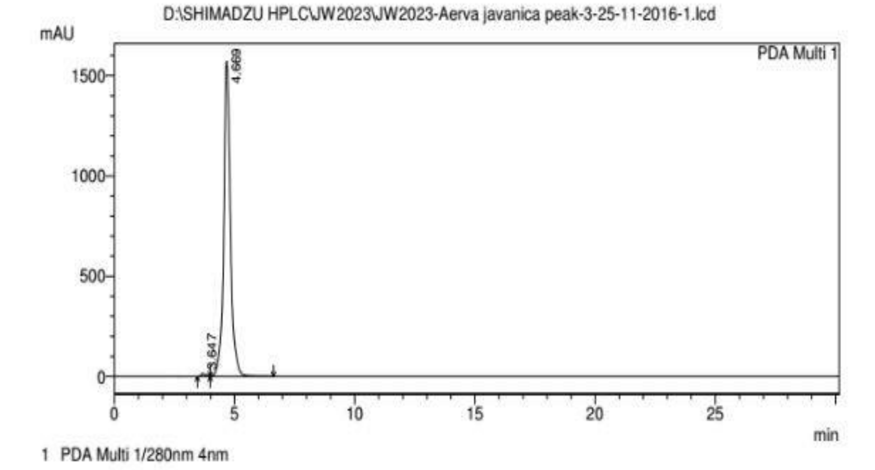
</td>
</tr>
<tr>
<td>Bruker FT-IR
Wavelength scan: 500-4000cm^{-1}</td>
<td>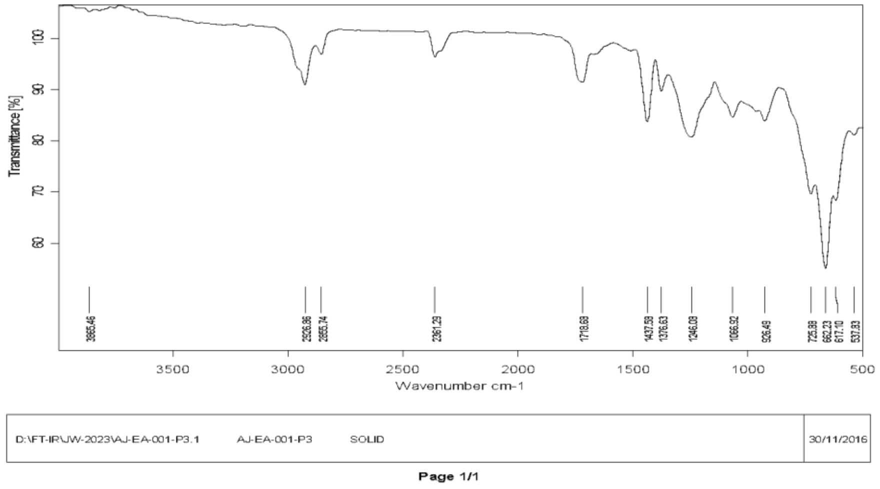
</td>
</tr>
<tr>
<td>BRUKER NMR 400MHz
$^{1}HNMR(CDCl_3)$:</td>
<td>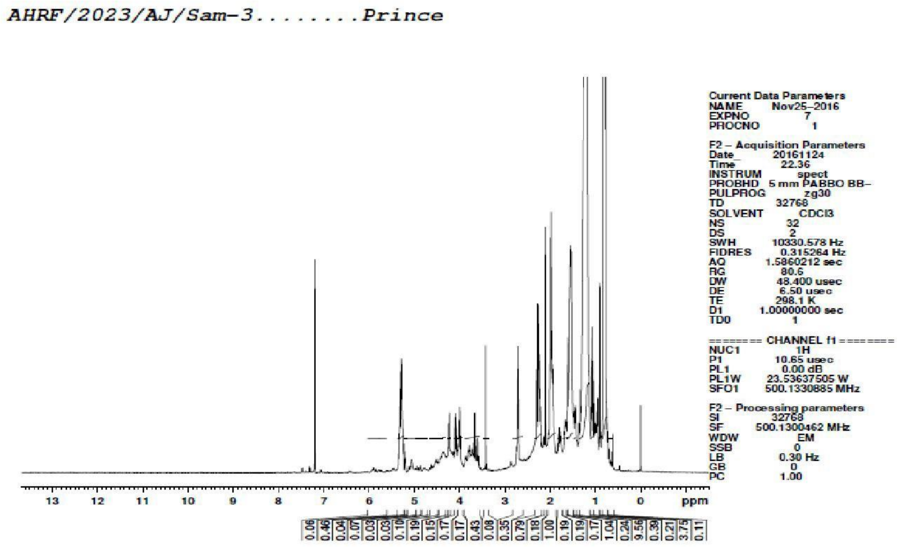
</td>
</tr>
</table>

BRUKER NMR 400MHz
13CNMR($CDCl_3$):

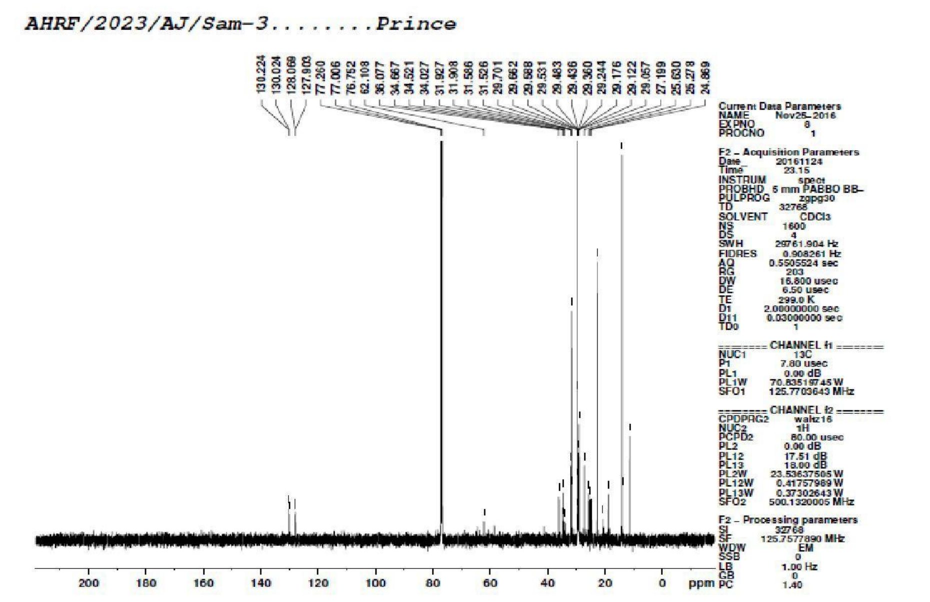

METHANOL EXTRACT

The methanol fraction were subjected to HPTLC and HPLC analysis. One pure compound was isolated and subjected to IR and NMR analysis for further identification.

COMPOUND 4 (AJ-ME-001-P3)

SPECIFICATON	SPECTRUM
Probable structure	Persinoside A and B 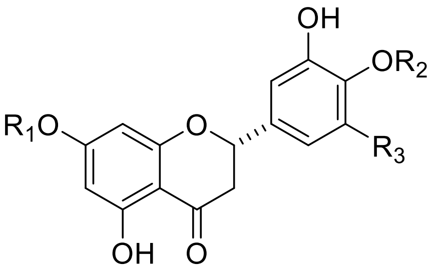 Persinoside A R_1=Me, R_2=Glc, R_3 = H<br>Persinoside B R_1=Glc, R_2=H, R_3 = OH
CAMAG HPTLC Scanner III<br>Mobile Phase: Hex: EtoAc (7:3)<br>Rf: 0.81<br>λ max: 352nm	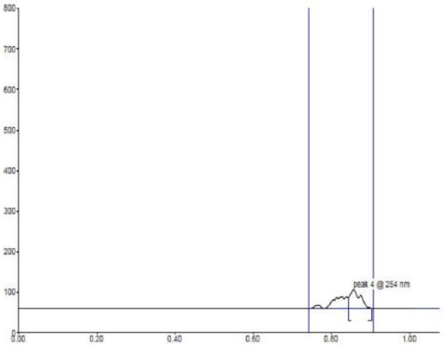 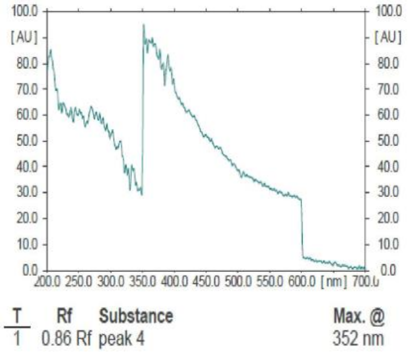

<table>
<tr>
<td>UV spectrum
PDA detector: 200-800nm
λ max: 195nm</td>
<td>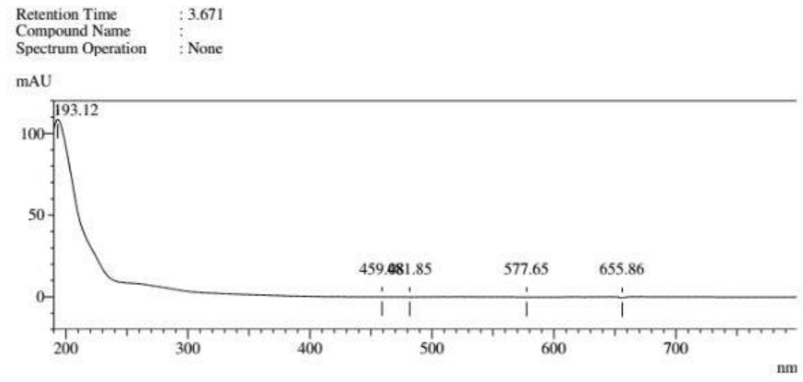
</td>
</tr>
<tr>
<td>SHIMADZU SPD-20A HPLC
Column: Enable C_{18}, 4.6x250mm (5micron)
Mobile Phase: ACN:H2O (15:85) with 0.1% H3PO4
λ max: 280nm
Inj. Vol: 20µl
Flow rate: 1.0ml/min
Rt: 3.671min</td>
<td>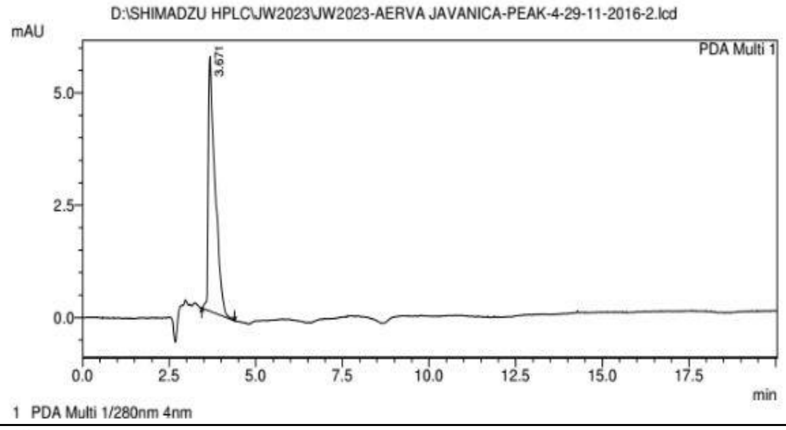
</td>
</tr>
<tr>
<td>Bruker FT-IR
Wavelength scan: 500-4000cm^{-1}</td>
<td>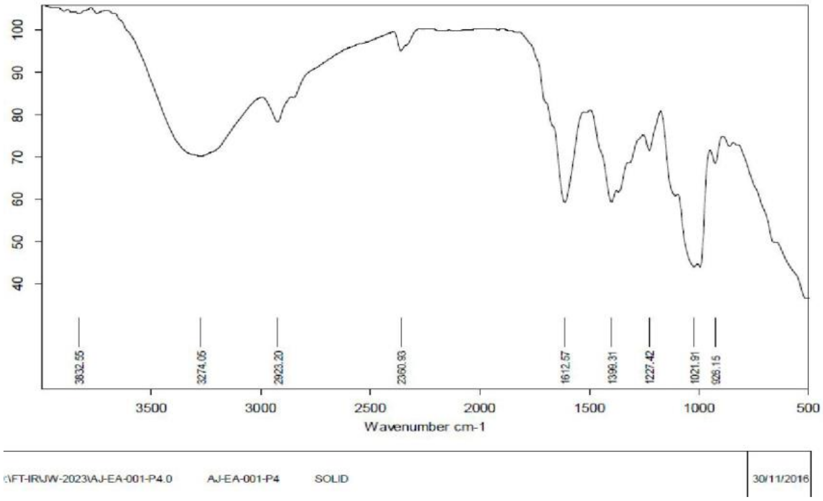
</td>
</tr>
</table>

BRUKER NMR 400MHz $^{1}HNMR(CDCl_3)$:	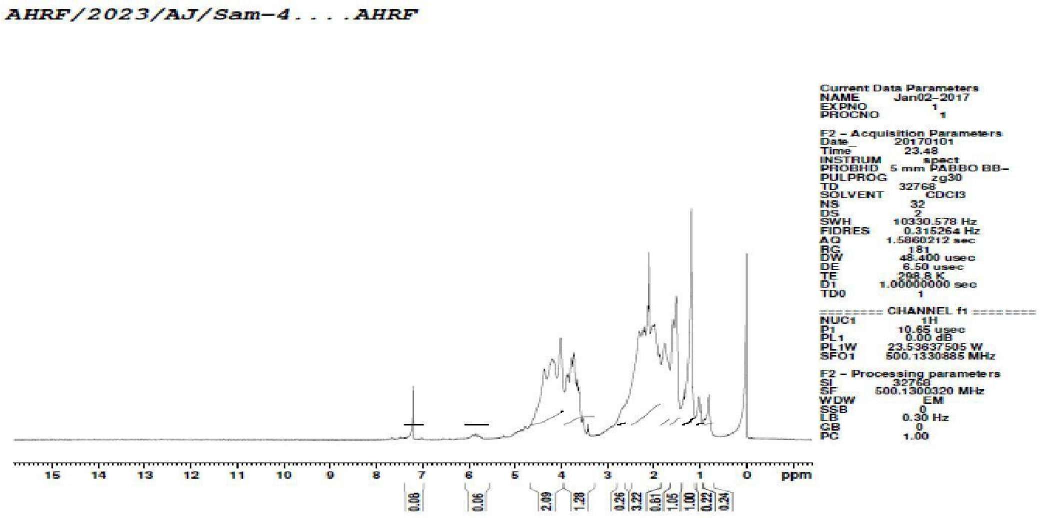
BRUKER NMR 400MHz $^{13}CNMR(CDCl_3)$:	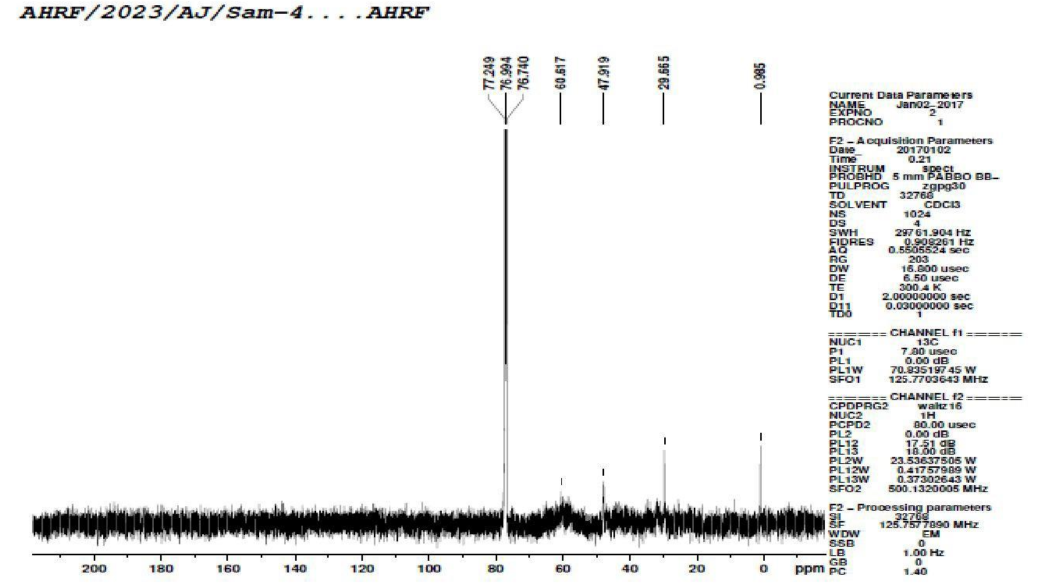
